# A multi-split mapping algorithm for circular RNA, splicing, trans-splicing and fusion detection

**DOI:** 10.1186/gb-2014-15-2-r34

**Published:** 2014-02-10

**Authors:** Steve Hoffmann, Christian Otto, Gero Doose, Andrea Tanzer, David Langenberger, Sabina Christ, Manfred Kunz, Lesca M Holdt, Daniel Teupser, Jörg Hackermüller, Peter F Stadler

**Affiliations:** 1 Junior Research Group Transcriptome Bioinformatics, Leipzig University, Haertelstrasse 16-18, Leipzig, Germany; 2 Interdisciplinary Center for Bioinformatics and Bioinformatics Group, University Leipzig, Haertelstrasse 16-18, Leipzig, Germany; 3 LIFE Research Center for Civilization Diseases, Leipzig University; 4 Department of Theoretical Chemistry, University of Vienna, Währinger Strasse 17, Vienna, Austria; 5 RNomics Group, Fraunhofer Institute for Cell Therapy and Immunology – IZI, Perlickstrasse 1, Leipzig, Germany; 6 Department of Dermatology, Venerology and Allergology, Leipzig University, Philipp-Rosenthal-Strasse 23, Leipzig, Germany; 7 Institute of Laboratory Medicine, Ludwig Maximilian University, Marchioninistrasse 15, Munich, Germany; 8 Young Investigators Group Bioinformatics and Transcriptomics, Department of Proteomics, Helmholtz Centre for Environmental Research – UFZ, Permoserstrasse 15, Leipzig, Germany; 9 Max Planck Institute for Mathematics in the Sciences, Inselstrasse 22, Leipzig, Germany; 10 Center for non-coding RNA in Technology and Health, University of Copenhagen, Grønnegårdsvej 3, Frederiksberg, Denmark; 11 Santa Fe Institute, 1399 Hyde Park Road, Santa Fe, NM, USA

## Abstract

Numerous high-throughput sequencing studies have focused on detecting conventionally spliced mRNAs in RNA-seq data. However, non-standard RNAs arising through gene fusion, circularization or trans-splicing are often neglected. We introduce a novel, unbiased algorithm to detect splice junctions from single-end cDNA sequences. In contrast to other methods, our approach accommodates multi-junction structures. Our method compares favorably with competing tools for conventionally spliced mRNAs and, with a gain of up to 40% of recall, systematically outperforms them on reads with multiple splits, trans-splicing and circular products. The algorithm is integrated into our mapping tool segemehl (http://www.bioinf.uni-leipzig.de/Software/segemehl/).

## Background

The term splicing refers to a post-transcriptional process in which the raw transcript (pre-mRNA) is cleaved from intronic DNA fragments. In general, the splicing mechanisms allow the recombination of protein-coding and non-coding RNA fragments and thus greatly increase the repertoire of potentially functional transcripts. While the overwhelming majority of splicing events occurs within the same pre-mRNA at consensus splice sites, some mRNAs are spliced at non-consensus sites. Many transcripts derived at non-consensus splice sites may have escaped detection in the past because of the assumptions built into the *in silico* analysis pipelines or due to the limited throughput of earlier RNA sequencing (RNA-seq) protocols.

Some species have developed mechanisms to fuse separately transcribed mRNAs. These mRNAs may stem from distant loci, opposite strands or homologous chromosomes. A prominent physiological example is the *mod(mdg4)* trans-splicing for *Drosophila melanogaster*[1]. Chimeric transcripts from different loci may be functional even in mammals [2]. Circular RNAs [3,4] are recognizable in RNA-seq data in the form of reads that contain apparent splice junctions that connect the end (start) of a split read fragment to the start (end) of a downstream (upstream) fragment. Very recently, they have been identified as an abundant class of regulatory transcripts functioning as microRNA sponges [5,6]. In addition to physiological trans-splicing, a number of transcripts potentially derived from the fusing of genes have been observed in different types of cancer, such as melanoma [7] and breast cancer [8]. In the following, we use for brevity the term ‘fusion transcript’ to refer to RNAs that stem from a fused gene or a trans-splicing event. Although trans-splicing and mRNA fusion events appear to be rare compared to the regular local and collinear splicing, fusion transcripts indicate potentially important functional entities or diagnostic marker genes. Their emerging importance mandates the use of analysis pipelines for RNA-seq data that ensure their efficient detection and inclusion in the subsequent data analysis workflow.

Several different algorithms for splice site detection have been devised so far. The original version of TopHat[9] predicts exon locations from the coverage data and attempts split read alignments across neighboring exons. This algorithm was not able to detect fusion events, so a new algorithm, TopHat-Fusion [10], was published and has since been integrated into TopHat2 along with some other modifications to the original algorithm. SpliceMap[11] starts by splitting the reads into fragments of 25 nucleotides and then attempts to align all fragments separately with a limited number of mismatches. Subsequently, canonical splice junctions are searched within a genomic interval of 400 Mb. The specificity of splice junctions may be improved by providing paired-end information. SpliceMap’s junction search is significantly distinct from TopHat2’s. The MapSplice algorithm [12] resembles SpliceMap. It also performs a segmentation of reads into tags and handles each tag individually. The tags are aligned to exons and junctions inferred from tags mapping to consecutive exons.

SplitSeek[13] also uses both the 5^′^ and 3^′^ ends of reads to infer spliced exons. SplitSeek does not make use of canonical splice site information and is not limited to a common locus. Another tool that was specifically designed for the detection of fusion transcripts, deFuse[14], makes use of paired-end information and triggers local alignments at positions of discordant paired-end reads.

Like TopHat2, SOAPsplice[15] is based on a Burrows-Wheeler transform and attempts to map the reads completely to the genome with no more than three mismatches or one gap. All unmapped reads are subsequently subjected to a split mapping with two segments. Each segment has to fulfill a number of quality criteria. GSNAP[16] uses hash tables to retrieve position lists and subsequently merges and filters them efficiently. It is able to allow for multiple mismatches and long indels and can detect short- and long-distance splicing. The RNA-seq mapper RUM[17] uses Bowtie[18] and BLAT[19] to detect annotated as well as *de novo* splice junctions. In a first step, reads are mapped against the genome as well as the user-supplied transcriptome. All unmapped reads are forwarded to BLAT and split alignments are merged. One of the latest tools for RNA-seq alignment, STAR[20], uses maximal mappable prefixes that are identified using suffix arrays. In a second step, the prefixes are ‘stitched’ together to reconstruct the isoforms. This algorithm was reported to be very fast – in fact it was shown to be more than 50 times faster than some of its competitors.

Here, we present a unified unbiased algorithm to detect splicing, trans-splicing and gene fusion events from single-end read data. The method, based on an enhanced suffix array, chaining and dynamic programming algorithms, is integrated into the mapping tool segemehl[21].

## Results and discussion

The algorithm presented here makes use of a read matching method with enhanced suffix arrays (ESAs) published earlier [21]. In brief, for a read of length *m*, the algorithm evaluates the best alignments with a limited number of mismatches, insertions and deletions for all 2(*m*−*ℓ*) suffixes of the read and its reverse complement, where *ℓ* is the minimum suffix length. An alignment qualifies as a seed if a score-based maximum E-value criterion and a maximum occurrence threshold are met. Subsequently, full reads will be aligned to all distinct seed locations in the reference genome using Myers’ semi-global bit-vector alignment [22]. All alignments passing a minimum accuracy threshold are reported. While the E-value, minimum accuracy and maximum occurrence parameter control the specificity and limit the number of multiple hits, the potentially large number of seeds from the beginning to the end of the read ensure a high sensitivity. For spliced or fusion transcripts, a successful semi-global alignment of the read is likely to fail. Instead, the ESA-based method will identify several seeds matching different locations or strands. The algorithmic strategy to identify splicing, trans-splicing or gene fusion sites is based on a greedy, score-based seed chaining followed by a Smith-Waterman-like transition alignment. This alignment optimizes the total score of a number of local alignments at different locations and strands. The algorithm does not have any effective length restrictions. Details are given in the Materials and methods section.

### Simulated data

The algorithm’s performance was compared with seven alternative split read methods: TopHat2, RUM, STAR, SOAPsplice, MapSplice, SpliceMap and GSNAP. In principle, all tools were run with default parameters for split-read mapping. Where available, fusion and trans-splice sensitive alignment parameters were turned on. With the exception of RUM, no extra annotations were given to any of the programs (see Materials and methods and Additional file 1).

To test the algorithm’s precision and sensitivity in all applicable scenarios, Illumina and 454 reads were simulated for regular splice junctions and a mixture of regular and non-regular splice junctions, i.e., splice junctions that connect opposite strands (strand-reversing) and splice junctions that connect distant exons (long-range splicing); see Materials and methods. The simulated Illumina and 454 reads were 100 bp and 400 bp long, respectively. Furthermore, we tested the recall on short circular transcripts (100 bp) as well as long linear and long circular reads of length 0.5 to 5 kB. The latter is the typical size range for circular transcripts in mammals. Short circularized RNAs are abundant in Archaea [23]. In accordance with other work in this field, Illumina and 454 error models were applied to the simulated reads. These models introduce mismatches, insertions and deletions to the reads to emulate sequencing artifacts (see Materials and methods).

**Figure 1:**
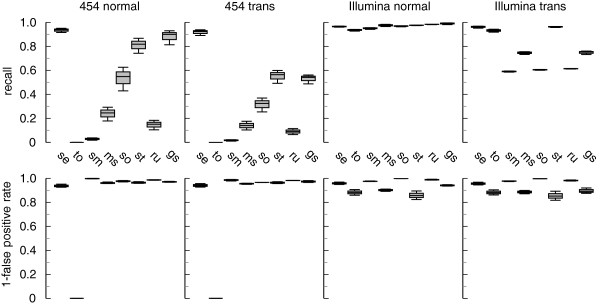
**Performance of various read aligners on simulated data sets with different splice events.** For simulated 454 reads (400 bp), segemehl performed significantly better in detecting conventional and ‘non-conventional’ (strand-reversing, long-range) splice junctions. segemehl was the only tool that consistently recalled more than 90% of conventional splice junctions. For ‘non-conventional’ splice events, segemehl extended its lead to 40% for recall without losing precision. Likewise, compared to three of the seven alternative tools, segemehl had a 30% increase in recall for irregularly spliced Illumina reads (100 bp). Compared to TopHat2, it had a slight increase while reporting significantly fewer false positives. At the same time, segemehl’s performance with simulated, regularly spliced Illumina reads was comparable with the other seven tools tested. gs, GSNAP; ms, MapSplice; ru, RUM; se, segemehl; sm, SpliceMap; so, SOAPsplice; st, STAR; to, TopHat2.

The results for simulated 454 and Illumina reads are summarized in Figure 1. segemehl performed best in both 454 simulations. In the data set with regular splice junctions, segemehl consistently recovered more than 90% of all simulated splice junctions. Its closest competitor, GSNAP, achieved a recall of between 81% and 92%. STAR was third, with less than 87% of recalled splice junctions. Probably due to length restrictions, TopHat2 did not report any results after running for over 1 week and was terminated. For irregular splice events, the difference was even more striking: while segemehl recovered approximately 90% of all simulated splice junctions, the next best competitor, STAR, achieved a recall of approximately 55%.

The improved performance for 454 reads did not significantly impair segemehl’s performance for Illumina data. All tools, including segemehl with a recall at the 95% level, found more than 90% of all simulated splice junctions. RUM, gaining an advantage by simultaneously aligning the reads to the genome and transcriptome, performed best in this scenario. The best genome-only aligner was STAR with a recall of 98%.

When trans-splicing events were included, five of the seven alternative tools recovered less than 80% of all splice junctions. TopHat2 had the best sensitivity and found more than 90% of the junctions. However, its false positive rate of more than 10% was quite high. In contrast, segemehl identified about 95% of all junctions in this data set and found only 2% false positives. For Illumina reads, the runtime of segemehl was comparable to most of the tools tested. Only MapSplice, GSNAP and STAR were significantly faster than segemehl (Additional file 1: Table S1).

In addition to these benchmarks with Illumina and 454 error models, we also wanted to investigate segemehl’s behavior for reads with higher error rates, for example caused by multiple single nucleotide variations or, more importantly, less successful sequencing runs. Therefore, we carried out further benchmarks with higher error rates (up to 5% mismatches and indels) and varying coverage. As expected, the recall increased with higher coverage and dropped with higher error rates. However, segemehl’s specificity was consistently at a very high level for all types of splice junctions (Additional file 1: Figure S1).

**Figure 2:**
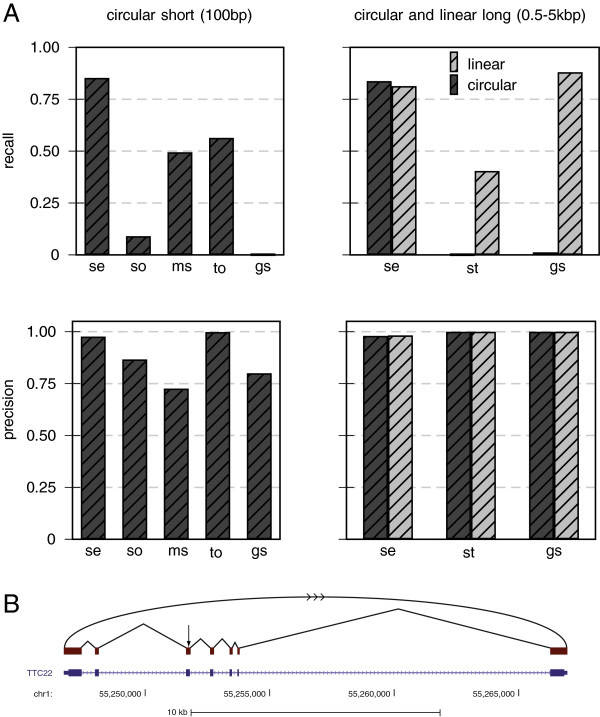
**Recall and precision for short circular, long circular and long collinear transcripts.** For this benchmark, we tested segemehl’s performance with sequence reads that were generated from the RefSeq database **(A)**. To simulate sequencing errors, we applied an Illumina error model to the short circular reads (100 bp) and a 454 error model to the long circular and collinear transcripts (0.5 to 5 kB). For short circular transcripts, segemehl achieved a recall of more than 85%, outcompeting all other tools while maintaining a high precision of 98%. Using RefSeq transcripts of length 0.5 to 5 kB, segemehl achieved a recall of more than 80% for circular and linear transcripts. Among the tools that were able to handle such long transcripts, segemehl was the only tool that was able to detect the circularization. For long collinear transcripts, GSNAP was slightly better than segemehl by 6%, at the expense of a nearly twofold increase in runtime (Additional file 1: Table S1). **(B)** The RefSeq TTC22 transcript is an example of a simulated circularization. The arrow indicates where the transcript has been artificially circularized. SpliceMap, RUM and STAR did not find any circular junctions (not shown). STAR and GSNAP were the only tools able to handle long reads. gs, GSNAP; ms, MapSplice; se, segemehl; so, SOAPsplice; st, STAR; to, TopHat2.

The performance for artificial short circular (100 bp), long circular and long linear reads (0.5 to 5 kb) is summarized in Figure 2. For the short reads, segemehl achieved a recall of 85% of all circular junctions using uniquely mapping split reads. The closest competitor, TopHat2, achieved a recall of 55%. SpliceMap, RUM and STAR did not find any circular junctions (Figure 2A). In contrast, STAR and GSNAP were the only tools that in principle were able to handle long reads. For linear transcripts, GSNAP was slightly ahead of segemehl by 6%, whereas STAR was merely able to recall 40% of all junctions. No circular junctions were recovered by STAR or GSNAP (Figure 2B).

### Real data

**Figure 3:**
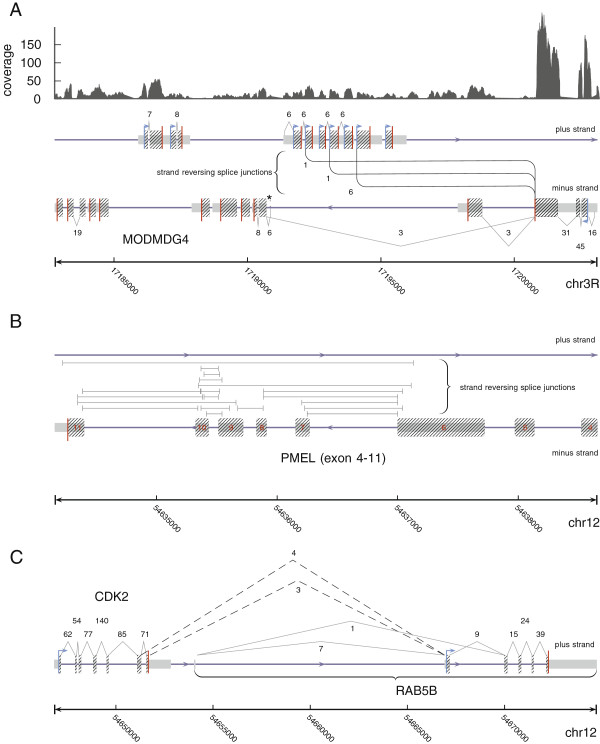
**Examples of (re-)discovered splicing events from single-end split reads.(A)** For *Drosophila melanogaster*, segemehl recovered three different previously described splice junctions linking the minus encoded exon three of *MODMDG4* on chromosome 3R to exons on the opposite strand. The strand-reversing splice junctions are annotated between the plus and minus strands. The direction of the strand-reversing splice junctions, i.e. from the minus to the plus strand, was inferred from annotation and prior knowledge. This was necessary because the RNA-seq library used was not strand specific. **(B)** For the human melanoma transcriptome data set, segemehl identified a very large number of strand-reversing splice junctions in the premelanosome protein (*PMEL*) gene locus. The split reads that support these junctions split from the plus strand to the minus strand and vice versa. Since we lack additional information, a direction for these junctions cannot be given. Only a selection of strand-reversing PMEL junctions is shown here. **(C)** For the same data set, segemehl found two alternative transcripts linking *CDK2* and *RAB5B* encoded on human chromosome 12. These junctions (dashed lines) are supported by split reads whose fragments map to the same strand, i.e. split reads that were not strand-reversing. Since the junctions exactly hit the annotated borders of the *CDK2* and *RAB5B* exons, we assigned them to the minus strand. chr, chromosome; *PMEL*, premelanosome protein.

We applied segemehl to a number of real data sets. Split-mapping of a *Drosophila melanogaster* RNA-seq data set resulted in the successful recovery of the previously described trans-splicing of the *MODMDG4* gene (Figure 3A) [1]. Most notably, segemehl revealed three alternative strand-reversing junctions consistent with a splice event between a common exon on the reverse strand to three exons encoded on the forward strand.

For a human melanoma transcriptome data set, the method identified the recently described *CDK2*-*RAB5B* read-through transcripts on chromosome 12 (cf. [7]) (Figure 3B). In addition, segemehl identified a huge number of strand-reversing split reads located at the locus of the premelanosome protein (*PMEL*). This gene is also known as Silver Homolog (*SILV*). This gene located on chromosome 12 encodes a melanocyte-specific transmembrane glycoprotein and is expressed under physiological conditions in melanosomes. It plays an essential role in the structural organization of premelansomes and has been suggested as a potential serum marker for melanoma (Figure 3C). More than 20% of the trans-splicing events detected in the sample occurred at this locus, making general errors in the RNA preparation and analysis highly unlikely. Thus, the PMEL locus might be an interesting target for further investigations.

**Figure 4:**
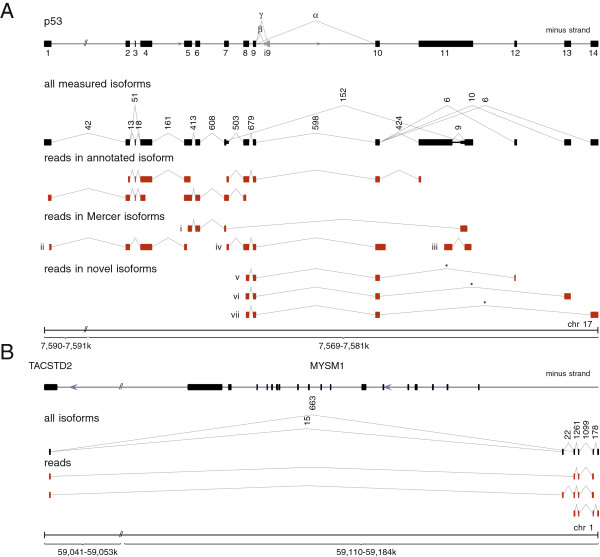
**Novel and known spliced transcript isoforms identified with long single-end 454 RNA-seq split reads.(A)** Transcript isoforms of the *p53* gene. In addition to previously reported isoforms, (i) to (iv) [24], we identified three novel canonically spliced isoforms, (v) to (vii). Consistent with [24], the *β* and *γ* isoforms were not expressed here. Each splice junction is labeled with its read support, i.e. the number of reads that map across this junction. For better comparability with [24], the *p53* gene, encoded on the minus strand of chromosome 17, is shown in the direction of transcription from left to right. The junctions marked with an asterisk have been experimentally validated. **(B)** Unannotated transcripts in the vicinity of the *TACSTD2* and *MYSM1* genes recovered from a HUVEC RNA data set [27]. segemehl revealed the exon structure of two novel transcript isoforms comprising at least four exons. One exon common to both isoforms was mapped to the *TACSTD2* gene. The associated introns enclose the *MYSM1* gene locus. The putative gene structure is supported by three exemplary multi-split reads (not strand-reversing). Some of the splice junctions have already been reported by ENCODE/CSHL (HUVEC polyA+ RNA-seq). The strandedness of the isoforms cannot be inferred.

We additionally applied our algorithm to a 454 data set published by [24] generated from RNA from human foot fibroblasts. As an example demonstrating the efficiency of segemehl, we considered the tumor suppressor gene *p53* (Figure 4A). This key regulator of the cell cycle is one of the most intensely studied genes because of its importance in cancer research. The functionally distinct variants and isoforms of *p53* have been the focus of an intense research effort [25,26]. Despite the attention this gene has already received, a reanalysis of the raw data [24] identified three previously unrecognized canonical splice variants. We have validated all three novel isoforms in venous fibroblasts using PCR and sequencing (see Materials and methods and Additional file 1). Since we failed to validate the *p53* isoforms in HUVEC cells (data not shown), these splice variants might be tissue-specific.

Novel transcripts were also predicted in a HUVEC 454 RNA-seq data set [27]. The example in Figure 4B shows two isoforms whose start is located anti-sense within the intronless *TACSTD2* gene. Their first intron contains on the opposite strand the entire *MYSM1* gene, which codes for a histone H2A deubiquitinase.

For a human prostate carcinoma cell line, we identified a transcript that aligns to adjacent regions on the plus and minus strands of the genome. We validated the occurrence of this split using RT-PCR followed by cloning and Sanger sequencing. Interestingly, the split was located in the 3^′^ UTR of the stearoyl-CoA desaturase gene *SCD* (Additional file 1 and Additional file 1: Figure S5), which has been implicated in prostate cancer [28].

For the RNA-seq data from HEK293 cells analyzed specifically for circular RNAs in [5], we were able to recover all circular RNAs that were experimentally validated by the authors of the original study (see Additional file 1).

Finally, we tested our algorithm on the transcriptome of the nematode *Caenorhabditis elegans*. The roundworm is known to have an extensive number of trans-spliced transcripts. In particular, spliced leader sequences (SLs) of about 22 nucleotides were spliced to the trimmed 5^′^ ends of many mRNAs [29]. The spliced leader sequences were encoded as part of small non-coding RNAs, the SL RNAs, in 28 loci scattered throughout the genome (Additional file 1: Table S3). To test whether SL trans-splicing can be detected directly from the segemehl mapping results, we reanalyzed a small part of the publicly available sequencing data generated by [30]. All trans-splicing junctions reported by the segemehl mapping (see Additional file 1) were required to have a minimum split read support of three. After masking rRNAs, we obtained approximately 9,000 junctions linking loci with a distance of more than 200 kb or on different chromosomes. These were supported by about 139,000 split reads. More than 90% of them connect to the genomic coordinates of the 3^′^ end of one of the annotated spliced leader sequences. This simple survey accurately reproduces results from a recent detailed analysis of *C. elegans* trans-splicing [31]. In particular we found that 70% of the ce6 mRNAs annotated in the UCSC genome browser were trans-spliced. We also recovered the expected distribution of spliced leader usage: SL1 is by far the most frequently used leader sequence (85.9% of all SL junctions) followed by SL2 (13%), SLf (0.4%), SLb (0.3%), SLc (0.2%), SLd (0.2%) and SLa (<0.1%) (Additional file 1: Table S5). Other trans-splice junctions found during this exercise are subject to further research.

To benchmark the speed of our split-read aligner, we aligned four different data sets with segemehl, STAR, SOAPsplice, GSNAP, TopHat2 and RUM. With the exception of STAR, in most scenarios segemehl was faster or on a par with the other tools tested (Additional file 1: Table S2). Because it uses a full ESA, the memory consumption of segemehl depends on the size of the reference genome (cf. [21]). Thus, it has the highest memory consumption among all tools tested. For large mammalian genomes, segemehl may not be feasibly applied on computers with less than 50 GB of memory. The size of the read library, however, does not affect the memory consumption. Note that for smaller genomes the memory consumption is considerably smaller, e.g. *C. elegans* 1.5 GB, *Drosophila melanogaster* 2.6 GB and *Arabidopsis thaliana* 1.8 GB.

## Conclusions

We have presented a novel algorithm for split-read mapping of single-end RNA-seq data that combines error-tolerant ESA-based seed mapping with a fast bit-vector alignment. It accommodates multiple splits within a single read and makes no *a priori* assumptions on the transcript structure. Implemented in the segemehl mapping tool, it readily identifies conventional splice junctions, collinear and non-collinear fusion transcripts, and trans-spliced RNAs, without the need for separate post-processing or an extensive computational overhead. Compared to widely used competitors, the method has significantly higher sensitivity and produces less false positives, especially for trans-splicing scenarios. This makes segemehl the method of choice for annotating rare transcript variants. Indeed, previously undescribed exons and additional splice junctions were readily identified.

Strikingly, the precision is maintained even for reads with higher error rates (Additional file 1: Figure S1). This feature is of particular interest when transcriptome data from organisms with high allelic variations are processed. It also makes it feasible to analyze transcriptome data by mapping to the genome of a closely related species as a reference.

Already the analysis of the few test data sets used here to verify the viability of our approach, shows that RNA-seq data sets readily contain evidence for a substantial number of transcripts with atypical structures. In addition to read-through transcripts, which preserve collinearity and can be explained by conventional splicing from an extended primary transcript, we also observed a moderate number of products that violate collinearity. These fall into at least three broad classes: strand-reversing RNAs, such as the fruit fly *MODMDG4* gene [1,32], that originate from both reading directions of a compact locus; permuted RNAs and circular isoforms [3-6,33,34] as for the *ANRIL* non-coding RNA [35]; and RNAs that are combined from components originating from different loci such as the rat *HongrES2* RNA [36] and several chimeric human proteins recently described in [37]. A few of these have been studied in some detail and at least in parts have also been characterized functionally [2]. These studies suggest that a part of the non-collinear transcriptome might be functional [38] and cannot be explained as a consequence of chromosomal rearrangements relative to the reference genome. An accurate mapping tool such as segemehl, which is sensitive to split reads and operates without an underlying model of valid transcript structure and hence does not discard non-collinear mapping results as artifacts, is therefore an indispensable tool for systematic investigations into this largely uncharted section of the transcriptome.

## Materials and methods

**Figure 5:**
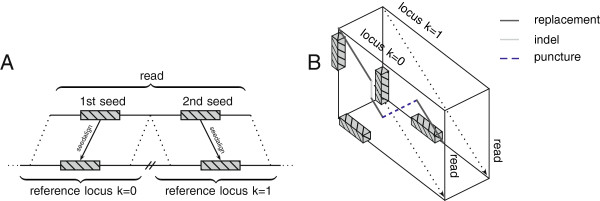
**A chain of seeds guides a local transition alignment across multiple genomic loci.** High-quality seeds mapping to different genomic loci, strands or chromosomes **(A)** are chained. Subsequently, the order of the seeds within the chain guides a walk through the alignment cube **(B)**. For each genomic locus, a local alignment with the read is performed. In addition to the regular Smith-Waterman recursions, the local transition alignment allows crossing between different reference loci.

The algorithmic strategy for split-read alignments is sketched in Figure 5.

### Chaining

After matching a read we obtain at most 2(*m*−*ℓ*) seed alignments. Each seed may have multiple occurrences in the genome. The start positions of each seed’s set of alignments in the reference genome are represented by the ESA interval [ *l*,*r*]. In addition to the aforementioned E-value and maximum occurrence parameters, the alignment seeds retrieved from the ESA are required to have a minimum Shannon entropy of 1.5. The Shannon entropy of a sequence *S* is defined by

(1) $$H(S)=-\overset{n-1}{\underset{i=0}{\sum }}p(s_{i})\underset{2}{\log}p(s_{i})$$

where *p*(*s*_*i*_) denotes the probability that the character *s*_*i*_ occurs in *S*. This additional prerequisite is necessary to drop low-complexity seeds caused, e.g., by poly-A tails or repeats that bypass the maximum occurrence threshold due to sequencing errors. In general, it cannot be ruled out that the Shannon entropy filter affects the detection of splice events in repetitive elements (cf. [39]). However, our calculations show that the majority of 20-bp and 40-bp windows in human ALU repeats have a Shannon entropy well above our threshold of 1.5 (see Additional file 1). Therefore, this filter does not impede the split-read mapping in ALU repeats per se. After passing the three filters, each alignment start of a seed in the reference genome is easily obtained in constant time using the suffix table of the ESA. Let  denote the set of seeds. In the chaining step, we aim to select an ordered chain of seeds c⊆S that optimally covers the read from start to end while at the same time maximizing the sum of alignment scores. Let *ψ* denote a function to obtain the alignment score of a seed and let *π*_*s*_ and *π*_*e*_ be two functions to determine a seed’s alignment start and end in the read, respectively. Finally, the score of a chain is evaluated using

(2) $$\sigma (c)=\overset{\left|c\right|}{\underset{k=1}{\sum }}\psi (c[\;k]\;)-\overset{\left|c\right|}{\underset{k=2}{\sum }}|\pi_{e}(c[\;k\;-\;1]\;)-\pi_{s}(c[\;k]\;)|$$

where c[k]∈S denotes the *k*th seed in chain *c*. In our implementation *ψ*(*c*[ *k*]) is the number of correctly matched nucleotides of fragment *i* minus the sum of mismatches, insertions and deletions in this fragment. A set of chains is obtained using a greedy chaining algorithm (Algorithm 1).

#### **Algorithm 1 **

Initially, seeds are sorted with respect to *π*_*s*_ and the sorted list is stored in *C*. In the first step, each single seed is a chain of its own. The computation proceeds by iterating over all chains in the list *C*. For each chain *C*_*i*_, the best preceding chain *c*^′^ is identified and concatenated with it. For two chains, *c*^′^ and *c*, the concatenation operator is denoted by *g*(*c*^′^,*c*).

It is easy to see that algorithm terminates after (|*C*|·(|*C*|−1))/2 iterations. Since there are at most 2(*m*−*ℓ*) seeds, the algorithm is of complexity *O*(*m*^2^).

### Local transition alignment

The chains are ranked with respect to their scores. To ensure a high precision, only the highest ranking chain is used in the subsequent alignment procedure. Furthermore, the chain by default should cover more than 80% of the read. As pointed out above, each seed might have multiple alignments across the genome. In this case segemehl selects those alignments that minimize the distance on the genome and, if possible, are on the same strand. Finally, for each seed we obtain exactly one position in the reference.

Guided by the chain of seeds, the local transition alignment algorithm maps the reads across multiple loci (Algorithm 2). A similar idea was independently proposed by [14]. Unlike McPherson *et al.*, we devised an algorithm that fully integrates the transition between multiple matrices to obtain an optimal local split alignment across different genomic loci. The local transition alignment method is a modification of the Smith-Waterman alignment.

#### **Algorithm 2 **

The algorithmic parts that realize the transition to other loci are marked by an asterisk. Note, that we have implemented the algorithm using lazy evaluation schemes. Furthermore, a penalty is applied to each transition in practice (not shown). Let *γ*(*c*[ *k*]) and *κ*(*c*[ *k*]) be functions that return the sequence and the strandedness for the reference locus to which the seed *c*[ *k*] was aligned, respectively. Note, that in practice the sequence returned by *γ* extends the exact alignment boundaries of *c*[ *k*] by several nucleotides to account for inaccuracies in segemehl’s seed-finding heuristics. The algorithm iterates over all seeds in the chain *c* and performs local alignments of the read *r* with their respective reference locus. Insertions and deletions are penalized with *δ*. *s*(*a*,*b*) scores matches and mismatches. The resulting alignment scores are stored in a three-dimensional matrix *M*. During the local alignment at *γ*(*c*[ *k*]), the algorithm keeps track of the last maximum score *lms*[ *k*,*i*] seen prior to the alignment of the *i*th character of the read. This additional table is the key to the local transition alignment algorithm. In addition to the local alignment recursions that maximize the score of *M*[ *i*,*k*,*j*], all *k*−1 preceding loci are checked for a possible transition using the *lms* table.

### Simulations and tools

To simulate both regular and irregular splice junctions, a sample of 10,000 isoforms was drawn from the ASTD database [40]. For the non-regular data set, 20% of the exons were either flipped to the opposite strand or substituted by a distant exon from the ASTD database. Any exon with a distance > 200 kB from the isoform or on a different chromosome was denoted as long-range splicing. The isoforms of each data set were extracted from the human genome (hg19) by concatenating their exon sequences. Using mason v.0.1.1 [41], we simulated Illumina and 454-like single-end reads of length 100 nucleotides and 400 nucleotides, respectively, from the isoforms of each data set with 10-fold, 15-fold and 20-fold coverage. The parameter values of the Illumina and 454 error model in mason were specified in accordance with the Bowtie2 paper [42]. For simulated Illumina reads, we used the default parameters; for 454, we used -k 0.3 -bm 0.4 -bs 0.1. Recall and false positive rates were calculated using the splice junctions predicted by each tool. Thus, the recall was calculated as the fraction of simulated junctions correctly identified by a tool. For each tool, the false positive rate was calculated by dividing the number of wrong junctions by the number of predicted junctions. For the comparisons we used TopHat2 (version 2.0.4), RUM (version 1.12_01), STAR (version 2.1.3e_r157), SOAPsplice (version 1.9), MapSplice (version 2.1.2), GSNAP (version 2013-11-27) and SpliceMap (version 3.3.5.2 (55)). All programs were run with default parameters for split-read mapping. With the exception of RUM, no additional annotation information was provided to any of the tools. Further details are given in Additional file 1.

### Split and isoform validation

To validate the newly identified canonical splice junctions in *p53*, we used RNA from venous fibroblasts. The RNA was isolated with TRIzol reagent (Life Technologies, Carlsbad, USA) and treated with RNase-free DNase (Qiagen, Hilden, Germany), according to the manufacturers’ instructions. The RNA samples were prepared in the context of another study recently published in PLoS Genetics [43]. The protocols for reverse transcription into cDNA are described therein.

PCR reactions were prepared in a final volume of 25 *μ*l using AmpliTaq Gold(R) 360 DNA Polymerase (Life Technologies, Carlsbad, USA) and primers were selected to span two exons to avoid co-amplification of genomic DNA: common forward primer (5^′^-CCGAGAGCTGAATGAGGCCTTG- 3^′^, 300 nM) and isoform-specific reverse primers (isoform (v) 5^′^-CATCACACTGCATACCTTGAATGTATGC- 3^′^; isoform (vi) 5^′^-CAGGCTAGAGTGCAATGGCGC- 3^′^; isoform (vii) 5^′^-GGCTCACGCCTGTAATCCCAGTAC- 3^′^; 300 nM each). The expected PCR product sizes were 322 bp, 213 bp and 178 bp for isoforms (v) to (vii), respectively. Cycling conditions were 95°C for 10 min and 40 three-step cycles of 95° for 20 s, 60°C (isoform (vi)) or 62°C (isoforms (v) and (vii)) for 30 s, and 72°C for 30 s. PCR products were subcloned using the TOPO TA Cloning Kit (Life Technologies, Carlsbad, USA) and sequencing reactions were performed with forward and reverse M13 primers (5 *μ*M, Life Technologies, Carlsbad, USA) and BigDye(R) Terminator v 3.1 chemistry (Life Technologies, Carlsbad, USA) according to the manufacturer’s instructions using an Applied Biosystems 3730xl DNA Analyzer.

### Data sets

Publicly available Illumina RNA-seq data sets of *D. melanogaster* [SRA:SRR166809] and a human melanoma sample [SRA:SRR018261-62] were downloaded from the National Center for Biotechnology Information short read archive. The 454 sequencing data for a human umbilical vein RNA-seq experiment [27] and RNA capture sequencing experiments of human fibroblasts [24] were retrieved from the Gene Expression Omnibus under [GEO:GSM951482] and [GEO:GSE29040]. The RNA-seq sample for HEK293 cells investigated in [5] was retrieved under [GEO:GSE43574]. Finally, we applied our algorithm to a *C. elegans* data set [SRA:SRX151602] to investigate RNA leader trans-splicing. All of these data sets are non-strand specific. In addition, we analyzed a strand-specific prostate cancer data set (see Additional file 1).

## Abbreviations

bp: Base pair; ESA: Enhanced suffix array; kb: Kilobase; Mb: Megabase; PCR: Polymerase chain reaction; RT: Reverse transcriptase; seq: Sequencing; SL: Spliced leader sequence; UTR: Untranslated region.

## Competing interests

The authors declare that they have no competing interests.

## Authors’ contributions

SH wrote the manuscript and developed the presented algorithm. CO co-developed the algorithm. SH, CO, GD, AT and DL performed the simulations and tested and verified the algorithm using simulated and real-life data. SC, JH, MK, LH and DT performed the wet-lab experiments. PFS designed and supervised the research. All authors read and approved the final manuscript.

## Supplementary Material

Additional file 1 **Supplementary benchmark.** Supplementary data on the parameters for all tools tested, the algorithms’ performance with real and simulated data and the results of wet-lab experiments. Click here for file
