# Supplementary material for: A multi-split mapping algorithm for circular RNA, splicing, trans-splicing and fusion detection
Source: Genome Biol. 2014 Feb 10;15(2):R34. doi: 10.1186/gb-2014-15-2-r34 (PMC4056463; doi:10.1186/gb-2014-15-2-r34)
Supplement: Additional file 1 — Supplementary benchmark. Supplementary data on the parameters for all tools tested, the algorithms’ performance with real and simulated data and the results of wet-lab experiments. [file gb-2014-15-2-r34-S1.pdf]

Supplemental material: A multi-split mapping algorithm for circular RNAs, splicing, trans-splicing, and fusion detection

Steve Hoffmann[1−3], Christian Otto[1−3], Gero Doose[1−3], Andrea Tanzer[4], David Langenberger[1−3], Sabina Christ[5], Manfred Kunz[6], Lesca M. Holdt[3,7], Daniel Teupser[3,7], Jörg Hackermüller[2,5,8] & Peter F. Stadler[2−4,9−11*]

[1] *Transcriptome Bioinformatics Group, University Leipzig, Germany.*
[2] *Interdisciplinary Center for Bioinformatics and Bioinformatics Group, University Leipzig, Germany.*
[3] *LIFE - Leipzig Research Center for Civilization Diseases, University Leipzig, Leipzig, Germany*
[4] *Department of Theoretical Chemistry, University of Vienna, Vienna, Austria.*
[5] *RNomics Group, Fraunhofer Institute for Cell Therapy and Immunology – IZI, Leipzig, Germany.*
[6] *Department of Dermatology, Venereology and Allergology, University Leipzig, Leipzig, Germany.*
[7] *Institute of Laboratory Medicine, Ludwig-Maximilians-University Munich, Munich, Germany*
[8] *Young Investigators Group Bioinformatics and Transcriptomics, Department Proteomics, Helmholtz Centre for Environmental Research – UFZ, Leipzig, Germany.*
[9] *Max-Planck-Institute for Mathematics in Sciences, Leipzig, Germany.*
[10] *Center for non-coding RNA in Technology and Health,University of Copenhagen, Denmark.*
[11] *Santa Fe Institute, Santa Fe, New Mexico, USA.*

Keywords: next generation sequencing, RNA-seq, multiple split read mapping, fusion transcripts

Running title: A multi-split read mapping algorithm

[*] *Corresponding author*: studla@bioinf.uni-leipzig.de

Supplementary Benchmarks

In the following we give additional benchmark data on the effect of errors and coverage on our tool (Supplementary Fig. 1), running times and memory usage on simulated and real data sets (Supplementary Tab.1, 2) as well as the exact parameters used for your evaluation.

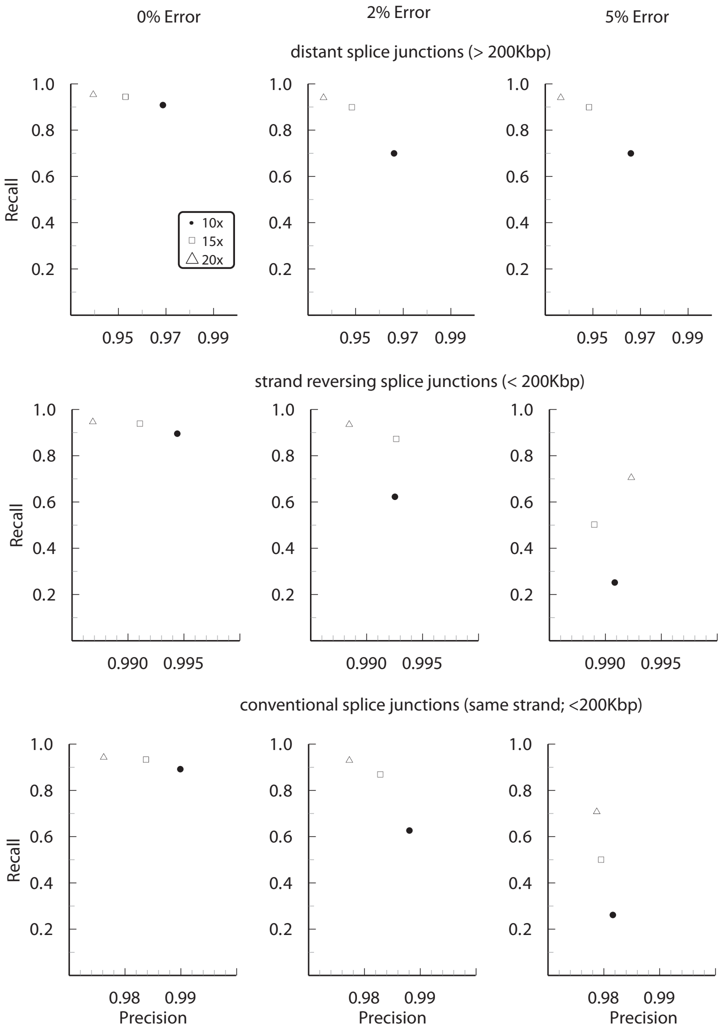

Figure 1: Effect of simulated sequencing errors and coverage on `segemehl` alignments in terms of recall and precision. Recall and precision were measured separately for long distant splice junctions (>200kb; top row), strand-reversing (middle row) and regular splice junctions (bottom row). In the graphs, coverage values (10-fold, 15-fold, 20-fold) are indicated as symbols. For each of these simulated splice junctions we simulated reads without errors (no mismatches and indels; left column), 2% error rate (middle column) and 5% error rate (right column). As expected, the recall increases with the coverage. Overall, segemehl shows a high precision. Interestingly, only the recall but not the precision declines when errors are introduced.

	segemehl	TopHat2	SpliceMap	MapSplice	SOAPsplice	STAR	RUM	GSNAP
				Illumina normal				
10x	1401.97	1908.83	1907.25	968.96	1935.35	25.37	1634.24	922.10
15x	2058.28	2630.04	2613.53	1357.54	2924.20	39.90	2384.61	1381.43
20x	2709.30	3356.13	3226.79	1766.17	3888.79	57.73	2872.59	1842.11
				Illumina trans				
10x	1466.49	1927.86	1983.13	1235.39	2249.18	26.32	1694.79	1436.51
15x	2122.52	2633.77	2602.47	1745.07	3360.47	41.26	2295.42	2123.29
20x	2790.97	3390.95	3293.05	2242.04	4475.16	61.26	3056.67	2819.13
				454 normal				
10x	2446.53	-	2627.13	937.89	5397.87	35.61	2105.31	1651.65
15x	3602.95	-	3642.76	1311.12	8088.81	53.64	2963.79	2524.77
20x	4882.83	-	4633.10	1688.17	10796.57	72.89	3923.66	3346.16
				454 trans				
10x	2548.14	-	2709.37	961.07	5629.92	33.50	2032.09	1983.12
15x	3764.13	-	3765.43	1357.22	8481.81	51.42	3023.82	2969.96
20x	5016.22	-	4825.93	1746.85	11232.61	69.46	3861.59	3926.48
				RefSeq circular short				
-	238.38	749.96	-	546.60	831.52	-	-	1359.54
				RefSeq circular long				
-	9643.94	-	-	-	-	486.64	-	16611.17
				RefSeq linear long				
-	9849.77	-	-	-	-	1668.61	-	17512.11

Table 1: User times for simulated reads for seven different split read aligners. In the case of Illumina reads the runtime of `segemehl` is comparable to most of the split read aligners tested here. `STAR` has the fastest runtime. `TopHat2` did not finish after 1 week with long 454-like reads.

	user time [hh:mm:ss]			
	SRR166809 [8]	SRR018261 [2]	SRR018262 [2]	SRR515313 [4]
segemehl	45:39:16	20:10:46	19:54:20	03:48:59
STAR	17:35:24	01:10:17	01:09:31	00:02:53
SOAPsplice	73:01:41	22:48:00	24:16:30	-
GSNAP	68:18:41	17:38:13	23:01:29	06:45:17
RUM	*	18:42:19	17:04:37	20:35:25
TopHat2	69:59:37	28:16:11**	28:58:29**	-
sequencer:	illumina	illumina	illumina	454
species:	drosophila	human	human	human
type:	paired end 100bp	paired end 50bp	paired end 50bp	single end
size:	4.6GB	1.6GB	1.6GB	197MB

Table 2: Runtime comparison of read aligners with different real life data sets. SOAPsplice and TopHat2 did not produce any output on 454 data sets. (*RUM has chrUextra in genome (output >200GB), **run with default parameters killed after 4 days; restarted with –no-coverage-search)

For the comparisons, we have used the following tools and options in all tested scenarios.

`GSNAP` (version 2013-11-27) [19],

```
>gsnap -A sam --nofails -D GSNAP -d hg19_gsnap -N 1 -t 15 file.fq
```

`MapSplice` (version 2.1.2) [18],

```
>python mapsplice.py -1 file.fq -c hg19/singlechroms/ -x hg19/ -Q fq --fusion-non-canonical --non-canonical -p 15 --qual-scale phred33
```

`RUM` (version 1.12_01) [7],

```
>RUM_runner.pl rum.config_hg19 file.fq outfolder 1 name
```

`segemehl` (version 0.1.5),

```
>segemehl.x -q file.fq -d hg19.fa -i hg19.idx -S -t 15 -s -o file.out
>testrealign.x -d hg19.fa -q file.out -n
```

`SOAPsplice` (version 1.9) [10],

```
>soapsplice -d hg19.fa.index -1 file.fq -o file.out -p 15
```

`SpliceMap` (version 3.3.5.2 (55)) [1],

```
>runSpliceMap SpliceMap.cfg (default, except num_threads = 15)
```

`STAR` (version 2.1.3e_r157) [5],

```
>STAR --genomeDir hg19_STAR/ --readFilesIn file.fq --runThreadN 15 --genomeLoad NoSharedMemory
```

`TopHat2` (version 2.0.4) [17, 11],

```
>tophat2 -p 15 -o outFolder --fusion-search bowtie/hg19 file.fq
```

For the long RefSeq reads, we used STAR following the supplement of [5].

`STAR` (version 2.3.1c; compiled as ‘STARlong’) [5],

```
>STAR --genomeDir hg19_STAR/ --readFilesIn file.fq --runThreadN 15 --outFilterMismatchNmax 100 --seedSearchLmax 30 --seedSearchStartLmax 30 \\
      --seedPerReadNmax 100000 --seedPerWindowNmax 100 --alignTranscriptsPerReadNmax 100000 --alignTranscriptsPerWindowNmax 10000 \\
      --chimSegmentMin 17 --chimScoreMin 17
```

Spliced leader trans-splicing in *C. elegans*

The *C. elegans* transcriptome data (SRX151602) was mapped using an increased accuracy of 95% (-A 95) and increased sensitivity for small splits (-Z 19) to the *C. elegans* reference genome ce6. Subsequently, we selected all split reads spanning more than 200K or split up between different chromosomes. After masking all split-reads starting or ending in the rRNA cluster on chrI:15060287-15072132 we found that more than 90% of all split-reads start or end in known spliced leader sequences (see Supplementary Tables 3, 4).

name	chromosome	strand	start	end	length
SL1	V	-	17122694	17122715	22
SL1	V	-	17123670	17123691	22
SL1	V	-	17124642	17124663	22
SL1	V	-	17125618	17125639	22
SL1	V	-	17129150	17129171	22
SL1	V	-	17130132	17130153	22
SL1	V	-	17131108	17131129	22
SL1	V	-	17428680	17428701	22
SL1	V	-	17121714	17121735	22
SL1	V	-	17120738	17120759	22
SL1	V	-	4573949	4573970	22
SL1	V	+	17118124	17118145	22
SL1	III	+	2430996	2431017	22
SL1	I	+	8781690	8781711	22
SL1	V	-	17128178	17128199	22
SL2	II	-	5544225	5544246	22
SL2	II	+	5543571	5543592	22
SL2	II	+	5844833	5844854	22
SL2	I	+	4172207	4172228	22
SLa	III	+	11090892	11090913	22
SLb	III	+	12250345	12250367	23
SLc	I	+	13297865	13297885	21
SLd	III	-	11090384	11090405	22
SLf	III	-	7140461	7140482	22
SLf	I	-	9055233	9055254	22
SLf	I	-	9057970	9057991	22
SLf	IV	+	5264896	5264917	22
SLg	IV	-	5317337	5317357	21

Table 3: Alignments of known spliced leader sequences [6, 15] to the ce6 genome assembly of *C. elegans*

	splice juncts.	trans split reads	% of trans split reads
known SL	6904	120998	68.1%
rRNA cluster on chrI	5358	38210	21.5%
RNA genes	207	1414	0.8%
others	2166	17016	9.6%

Table 4: Trans-splice junctions involving SL and rRNA loci. The majority of all split reads spanning more than 200K or split up between different chromosomes are aligned to known spliced leader positions or rRNAs. The rRNA cluster on chrI accumulates 21.5% of the trans-split reads, while the rest of RNA genes, including rRNAs outside the cluster on chrI, account for less than one percent of trans split reads.

leader	splice juncts.	dist split reads	% SL juncts.	% SL dist split reads
SL1	5929	109064	85.9%	90.1%
SL2	895	11484	13.0%	9.5%
SLa	2	10	<0.1%	<0.1%
SLb	21	116	0.3%	0.1%
SLc	16	72	0.2%	0.1%
SLd	12	66	0.2%	0.1%
SLf	29	186	0.4%	0.1%

Table 5: SL usage. The spliced leader SL1 is the most frequently used spliced leader as determined by our split read alignment.

Validation of novel p53 isoforms

Applying our split read algorithm to the data of [14] (GSE29040) we predicted the following novel isoforms:

```
>p53_isoform_v
CCGAGAGCTGAATGAGGCCTTGGAACTCAAGGATGCCCAGGCTGGGAAGGAGCCAGGGGG
GAGCAGGGCTCACTCCAGGGAATGCCAAACACTCTCCCCAGGAGATCCAGACCCGCCTCT
TTCAGAGACTTTTAACTTAAACATCTGTCCCTACCCAGCAGGCAAACTAGAGCTCCTGAA
GCTCAGTCCCTGTCCTTGCCTCTGTAGACAGGTCACCTTGATGAGCTTCCTTTTTTTTTT
TTTAATTTTTTTTTATTTTAGGCTTTATTGGGGCATAATTGATCCCCCAAAATTGCATAC
ATTCAAGGTATGCAGTGTGATG
>p53_isoform_vi
CCGAGAGCTGAATGAGGCCTTGGAACTCAAGGATGCCCAGGCTGGGAAGGAGCCAGGGGG
GAGCAGGGCTCACTCCAGAAAACATTTCCGGCCAGGCACGGTGGATCACACCTGTAATCC
CAGCTACTCGGGAGGCTGAGGCAGGAGAATCGCTTGAACCCAGGAGGCAGAGGGTTGCAG
TGAGCCGAGATTGCGCCATTGCACTCTAGCCTG
>p53_isoform_vii
CCGAGAGCTGAATGAGGCCTTGGAACTCAAGGATGCCCAGGCTGGGAAGGAGCCAGGGGG
GAGCAGGGCTCACTCCAGAGACAGGGTTTCACCGTGTTAGCCAGGATGGTCTCGATCTCC
TGACCTCGTGATCCGCCAGCCTTGGCCTCCTAAAGTACTGGGATTACAGGCGTGAGCC
```

For the validation of the newly identified canonical splice junctions in p53 we used RNA from venous fibroblasts. The RNA was isolated with TRIzol reagent (Life Technologies) and treated with RNase-free DNase (Qiagen) according to the manufacturers' instructions. The RNA samples were prepared in the context of another study recently published in PLoS Genetics [9]. The protocols for reverse transcription into cDNA are described therein. PCR reactions were prepared in a final volume of 25 μl using AmpliTaq Gold(R) 360 DNA Polymerase (Life Technologies) and primers were selected to span two exons in order to avoid co-amplification of genomic DNA. We used a common forward primer and three isoform-specific reverse primers. (Fig. 2).

`common forward primer: 5'-CCGAGAGCTGAATGAGGCCTTG-3', 300nM`

```
isoform v reverse primer: 5'-CATCACACTGCATACCTTGAATGTATGC-3', 300nM
isoform vi reverse primer: 5'-CAGGCTAGAGTGCAATGGCGC-3', 300nM
isoform vii reverse primer 5'-GGCTCACGCCTGTAATCCCAGTAC-3', 300nM
```

Expected PCR product sizes were 322bp, 213bp, and 178bp for isoform v-vii, respectively. Cycling conditions were 95°C for 10 minutes and 40 three-step cycles of 95° for 20 seconds, 60°C (isoform vi) or 62°C (isoform v/vii) for 30 seconds, and 72°C for 30 seconds. PCR products were subcloned using the TOPO TA Cloning Kit (Life Technologies) and sequencing reactions were performed with forward and reverse M13 primers (5μM, Life Technologies) and BigDye(R) Terminator v 3.1 chemistry (Life Technologies) according to the manufacturer's instructions an Applied Biosystems 3730xl DNA Analyzer.

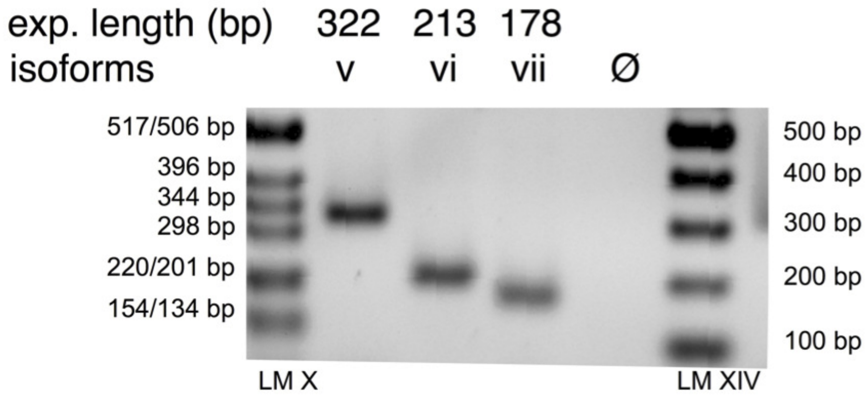

Figure 3: PCR validation of three p53 novel isoforms on 2% LMP agarose gel. Observed product sizes of PCR products were as expected. (LM X: Roche DNA Molecular Weight Marker X (0.07- 12.2 kbp); LM XIV: Roche DNA Molecular Weight Marker XIV (100-1500bp))

The following unclipped sequences contain the cloned PCR product along with the pCR 2.1 TOPO TA cloning sequences. The PCR products are given in uppercase.

```
> p53-isoform_v-M13_fwd_primer -- unclipped
ggccttcctcctatagggcgattgggccctctagatgcatgcttcgagcggccgccagtg
tgatggatatctgcagaattcgcccttCATCACACTGCATACCTTGAATGTATGCAATTT
TGGGGGATCAATTATGCCCCAATAAAGCCTAAAATAAAAAAAAAATTAAAAAAAAAAAAGGA
AGCTCATCAAGGTGACCTGTCTACAGAGGCAAGGACAGGGACTGAGCTTCAGGAGCTCTA
GTTTGCCTGCTGGGTAGGGACAGATGTTTAAGTTAAAAGTCTCTGAAAGAGGCGGGTCTG
GATCTCCTGGGGAGAGTGTTTGGCATTCCCTGGAGTGAGCCCTGCTCCCCCCTGGCTCCT
TCCCAGCCTGGGCATCCTTGAGTTCCAAGGCCTCATTCAGCTCTCGGaagggcgaattcc
agcacactggcggccgttactagtggatccgagctcggtaccaagcttgatgcatagctt
gagtattctatagtgtcacctaaatagcttggcgtaatcatggtcatagctgtttcctgt
gtgaaattgttatccgctcacaattccacacaacatacgagccggaagcataaagtgtaa
agcctggggtgcctaatgagtgagctaactcacattaattgcgttgcgctcactgcccgc
```

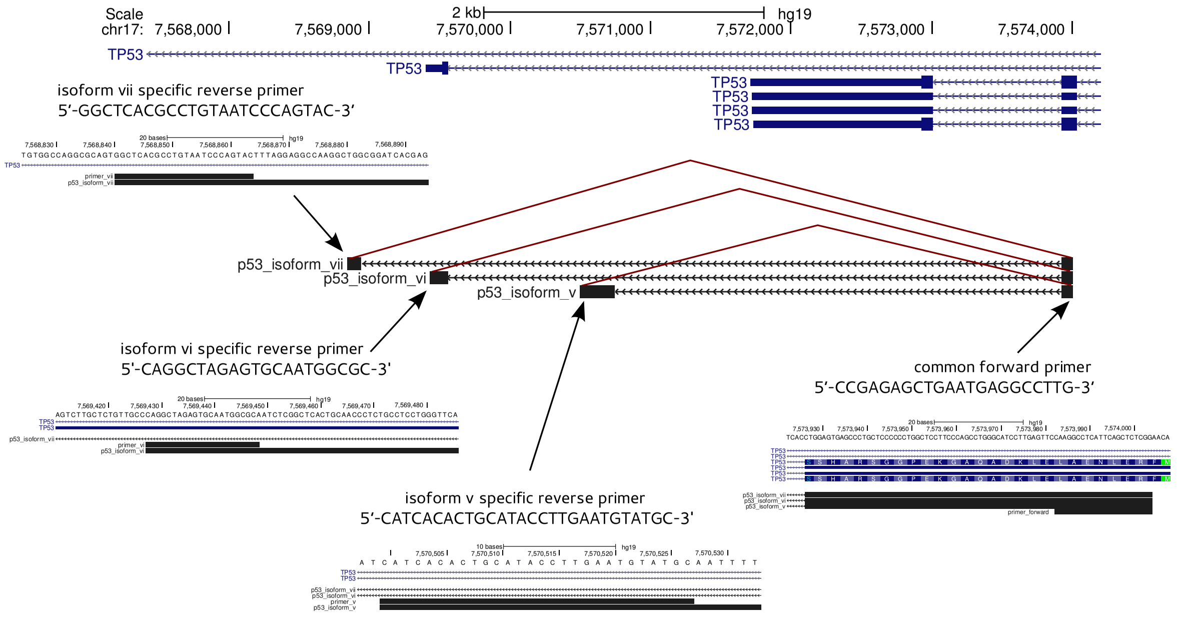

Figure 2: PCR primers for the validation of the novel p53 isoforms detected by segemehl. We used a common forward primer and three isoform specific reverse primers.

tttccagtcgggaaacctgtcgtgccagctgcattaatgaatcggccaacgcgcggggag
aggcggtttgcgtattgggcgctcttccgcttcctcgctcactgactcgctgcgctcggt
cgttcggctgcggcgagcggtatcagctcactcaaaggcggtaatacggttatccacaga
atcaggggataacgcaggaaagaacatgtgagcaaaaggccagcaaaggccaggaaccgt
aaaaaggccgcgttgctggcgtttttccataggctccgcccccctgacgagcatcacaaaat
cgacgctcaagtcagaggtggcgaaacccgacaggactataaagataccaggcgtttccc
cctggaagctccctcgtgcgctctcctgttccgaccctgccgcttaccggatacctgtccgcttt
ctcctcggagcgtggcgctttctcatagctcacgctgtagtttctcagtcgtgtagtcgt
tcgcttcaggctgcctgtgacgacctccgtcaggcgaccgctgcggccttatccgtacta
tcgtcttgagtccacccgtagacc
> p53-isoform_v-M13_rev_primer -- unclipped
tggggggtgccattaggtgaactatagaatactcaagctatgcatcaagcttggtaccgag
ctcggatccactagtaacggccgccagtgtgctggaattcgcccttCCGAGAGCTGAATG
AGGCCTTGGAACTCAAGGATGCCCAGGCTGGGAAGGAGCCAGGGGGGAGCAGGGCTCACT
CCAGGGAATGCCAAACACTCTCCCCAGGAGATCCAGACCCGCCTCTTTCAGAGACTTTTA
ACTTAAACATCTGTCCCTACCCAGCAGGCAAACTAGAGCTCCTGAAGCTCAGTCCCTGTC
CTTGCCTCTGTAGACAGGTCACCTTGATGAGCTTCCTTTTTTTTTTTAATTTTTTTTTAT
TTTAGGCTTTATTGGGGCATAATTGATCCCCCAAAATTGCATACATTCAAGGTATGCAGT
GTGATGaagggcgaattctgcagatatccatcacactggcggccgctcgagcatgcatct
agagggcccaattcgccctatagtgagtcgtattacaattcactggccgtcgttttacaa
cgtcgtgactgggaaaaccctggcgttacccaacttaatcgccttgcagcacatcccccct
ttcgccagctggcgtaatagcgaagaggcccgcaccgatcgcccttcccaacagttgcgc
agcctgaatggcgaatggacgcgccctgtagcggcgcattaagcgcggcgggtgtggtgg
ttacgcgcagcgtgaccgctacacttgccagcgccctagcgcccgctcctttcgctttct
tcccttcctttctcgccacgttcgccggctttccccgtcaagctctaaatcgggggctcc
ctttagggttccgatttagtgctttacggcacctcgaccccaaaaaacttgattagggtg
atggttcacgtagtgggccatcgccctgatagacggtttttcgccctttgacgttggagt
ccacgttctttaatagtggactcttgttccaaactggaacaacactcaaccctatctcggtc
tattcttttgatttataagggattttgccgatttcggcctattggttaaaaaatgagctgatttaa
caaaaattacggcgatttaacaaaatcaggcgcaaggctgctaagagcgaacaccgtagaa
gcagtcgcagaacgtgctgacctgatgagtcactactgcatctgcaagaaaatcaagcta
agaagcaggtacctgcatggcat
> p53-isoform_vi-M13_fwd_primer -- unclipped
cggatttacccttatagggggcgaattgggccctctagatgcatgctcgagcggccgccag
tgtgatggatatctgcagaattcgcccttCAGGCTAGAGTGCAATGGCGCAATCTCGGCT
CACTGCAACCCTCTGCCTCCTGGGTTCAAGCGATTCTCCTGCCTCAGCCTCCCGAGTAGC
TGGGATTACAGGTGTGATCCACCGTGCCTGGCCGGAAATGTTTTCTGGAGTGAGCCCTGC
TCCCCCCTGGCTCCTTCCCAGCCTGGGCATCCTTGAGTTCCAAGGCCTCATTCAGCTCTC
GGaagggcgaattccagcacactggcggccgttactagtggatccgagctcggtaccaag
cttgatgcatagcttgagtattctatagtgtcacctaaatagcttggcgtaatcatggtc
atagctgtttcctgtgtgaaattgttatccgctcacaattccacacaacatacgagccgg
aagcataaagtgtaaagcctggggtgcctaatgagtgagctaactcacattaattgcgtt
gcgctcactgcccgctttccagtcgggaaacctgtcgtgccagctgcattaatgaatcgg
ccaacgcgcggggagaggcggtttgcgtattgggcgctcttccgcttcctcgctcactga
ctcgctgcgctcggtcgttcggctgcggcgagcggtatcagctcactcaaaggcggtaat
acggttatccacagaatcaggggataacgcaggaaagaacatgtgagcaaaaggccagca

aaaggccaggaaccgtaaaaaggccgcgttgctggcgtttttccataggctccgcccccc
tgacgagcatcacaaaaatcgacgctcaagtcagaggtggcgaaacccgacaggactata
aagataccaggcgtttccccctggaagctccctcgtgcgctctcctgttccgaccctgcc
gcttaccggatacctgtccgcctttctcccttcgggaagcgtggcgctttctcatagctc
acgctgtaggtatctcagttcggtgtaggtcgttcgctccaagctgggctgtgtgcacgaacc
ccccgttcagcccgaccgctgcgccttatccggtaactatcgtcttgagtccaacccggtaag
acacgacttatcgccactggcagcagccactggtaacaggattagcagagcgaggtatg
agccggtgcctaccgaagatcttgagatgtgggccctactt
> p53-isoform_vi-M13_rev_primer -- unclipped
agtgggtgcattttagggtgacactatagaatactcaagctatgcatcaagcttggtacc
gagctcggatccactagtaacggccgccagtgtgctggaattcgcccttCCGAGAGCTGA
ATGAGGCCTTGGAACTCAAGGATGCCCAGGCTGGGAAGGAGCCAGGGGGGAGCAGGGCTC
ACTCCAGAAAACATTTCCGGCCAGGCACGGTGGATCACACCTGTAATCCCAGCTACTCGG
GAGGCTGAGGCAGGAGAATCGCTTGAACCCAGGAGGCAGAGGGTTGCAGTGAGCCGAGAT
TGCGCCATTGCACTCTAGCCTGaagggcgaattctgcagatatccatcacactggcggcc
gctcgagcatgcatctagagggcccaattcgccctatagtgagtcgtattacaattcact
ggccgtcgttttacaacgtcgtgactgggaaaaccctggcgttacccaacttaatcgcct
tgcagcacatccccctttcgccagctggcgtaatagcgaagaggcccgcaccgatcgccc
ttcccaacagttgcgcagcctgaatggcgaatggacgcgccctgtagcggcgcattaagc
gcggcgggtgtggtggttacgcgcagcgtgaccgctacacttgccagcgccctagcgccc
gctcctttcgctttcttcccttcctttctcgccacgttcgccggctttccccgtcaagct
ctaaatcgggggctccctttagggttccgatttagtgctttacggcacctcgaccccaa
aaaacttgatttagggtgatggttcacgtagtgggccatcgccctgatagacggtttt
cgccctttgacgttggagtccacgttctttaatagtggactcttgttccaaactggaac
aacactcaaccctatctcggtctattcttttgatttataagggattttgccgatttcggc
ctattggttaaaaaatgagctgatttaacaaaaatttaacgcgaattttaacaaaatattaacgc
aggctgcctaaggaagccgaacatgtagaagccaagtctgcagaaacggtggcttga
> p53-isoform_vii-M13_fwd_primer -- unclipped
gccccggggcaattatagggcgaattgggccctctagatgcatgctcgagcggccgcca
gtgtgatggatatctgcagaattcgcccttGGCTCACGCCTGTAATCCCAGTACTTTAGG
AGGCCAAGGCTGGCGGATCACGAGGTCAGGAGATCGAGACCATCCTGGCCAACATGGTGA
AACCCTGTCTCTGGAGTGAGCCCTGCTCCCCCCTGGCTCCTTCCCAGCCTGGGCATCCTT
GAGTTCCAAGGCCTCATTCAGCTCTCGGaagggcgaattccagcacactggcggccgtta
ctagtggatccgagctcggtaccaagcttgatgcatagcttgagtattctatagtgtcac
ctaaatagcttggcgtaatcatggtcatagctgtttcctgtgtgaaattgttatccgctc
acaattccacacaacatacgagccggaagcataaagtgtaaagcctggggtgcctaatga
gtgagctaactcacattaattgcgttgcgctcactgcccgctttccagtcgggaaacctg
tcgtgccagctgcattaatgaatcggccaacgcgcggggagaggcggtttgcgtattggg
cgctcttccgcttcctcgctcactgactcgctgcgctcggtcgttcggctgcggcgagcg
gtatcagctcactcaaaggcggtaatacggttatccacagaatcaggggataacgcagg
aaagaacatgtgagcaaaaggccagcaaaaggccaggaaccgtaaaaaggccgcgttgct
ggcgtttttccataggctccgcccccctgacgagcatcacaaaaatcgacgctcaagtca
gaggtggcgaaacccgacaggactataaagataccaggcgtttccccctggaagctccctc
gtgcgctctcctgttccgaccctgccgcttaccggatacctgtccgcctttctcccttcgg
gaagcgtggcgctttctcatagctcacgctgtaggtatctcagttcggtgtaggtcgttc
gctccaagctgggctgtgtgcacgaaccccccgttcagcccgaccgctgcgccttatccgg

```
tcggtaacttattgtcttgagtccaacccggtaagacaccgactatcgccactgcagcag
cacttgtaacagatttagccaagcgaggcattgatggcggctacagagctcttgaagtgg
gtgcctactctacgcttacaccttagaaaactgatttgcaaactcgtcgcctccgtcgaa
agccccgatac
> p53-isoform_vii-M13_rev_primer -- unclipped
tggggatgcctttttaggtgaactatagaatactcaagctatgcatcaagcttggtaccg
agctcggatccactagtaacggccgccagtgtgctggaattcgcccttCCGAGAGCTGAA
TGAGGCCTTGGAACTCAAGGATGCCCAGGCTGGGAAGGAGCCAGGGGGGAGCAGGGCTCA
CTCCAGAGACAGGGTTTCACCATGTTGGCCAGGATGGTCTCGATCTCCTGACCTCGTGAT
CCGCCAGCCTTGGCCTCCTAAAGTACTGGGATTACAGGCGTGAGCCaagggcgaattctg
cagatatccatcacactggcggccgctcgagcatgcatctagagggcccaattcgcccta
tagtgagtcgtattacaattcactggccgtcgttttacaacgtcgtgactgggaaaaccc
tggcgttacccaacttaatcgccttgcagcacatccccctttcgccagctggcgtaatag
cgaagaggcccgcaccgatcgcccttcccaacagttgcgcagcctgaatggcgaatggac
gcgccctgtagcggcgcattaagcgcggcgggtgtggtggttacgcgcagcgtgaccgct
acacttgccagcgccctagcgcccgctcctttcgctttcttcccttcctttctcgccacg
ttcgccggctttccccgtcaagctctaaatcgggggctccctttagggttccgatttagt
gctttacggcacctcgaccccaaaaaacttgattagggtgatggttcacgtagtgggcca
tcgccctgatagacggtttttcgccctttgacgttggagtccacgttctttaatagtgga
ctcttgttccaaactggaacaacactcaaccctatctcggtctattcttttgatttataa
gggattttgccgatttcggcctattggttaaaaatgagctgatttaacaaaaatttacgcg
aattttaacaaaatttcaggcgcaagggctgctaaaggaagcggacacgtagaaagccag
tccgccagaaacggtgctgacccggatgatgtcagctactgggcttatcttggacaggga
aacgcagcgcaaagagaaagcagtagcttgcaatgggcttacattgcgaatagctagaac
ttgggcggatttatggacagcaggcaactgaattgcaagcttgggcgccctctggtaggt
ggagccttgcagtactgatgcttcttgcacagatctgatgcccaagagaattaagattct
gtattcagcga
```

Shannon entropy in ALU repeats and circular transcripts

To investigate whether our seed selection procedure and especially the Shannon entropy filter prohibits the detection of circular transcripts in ALU elements, we have calculated the median entropy in 20bp and 40bp windows for each ALU repeat, annotated in hg19 (Fig. 4). Within all ALUs, the smallest median entropy we measured was 1.785. Thus, our results indicate that the required minimum shannon entropy of 1.5 does not impede the split-read mapping procedure of `segemehl`.

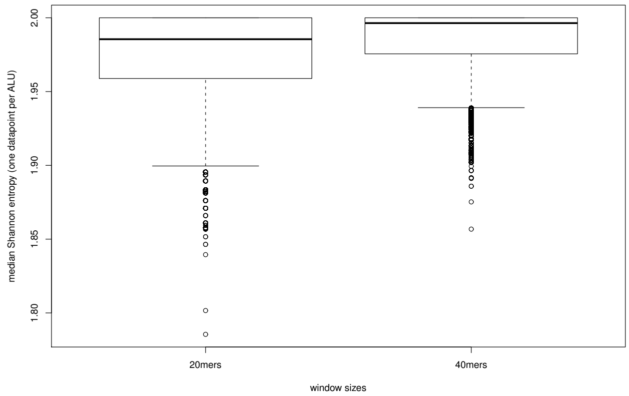

Figure 4: Median entropy of ALU repeats in 20bp and 40bp windows. Most of the ALUs have a median shannon entropy of >1.8, i.e. half of the windows calculated in these ALUs have a shannon entropy >1.785.

To check whether segemehl is indeed able to map successfully onto ALU repeats, we have extracted the sequences of all Human ALU repeats (∼1.1 Million) that were annotated in the RepeatMasker track of hg19. The ALU sequences were artificially circularized and reads of length 100nt were generated. Subsequently, the reads were mapped to the Human genome using `segemehl`. In total, 99% of the reads were aligned by our tool. Most of the aligned reads (54.5%) were aligned without a split (end-to-end), while only 35.5% of them contained a circular junction. This can easily be explained by the fact that ALU repeats are frequently found in multiple adjacent copies in the genome. By design, `segemehl` operates conservative and attempts to map any read collinearly if possible.
Since 99% of the reads could be mapped, seed and alignment quality filters do not per se impede the mapping of ALU transcripts. However, the above result indicates that a circular transcript could be missed if it was also explainable by a collinear transcript. Obviously, the information of the read is simply not sufficient, e.g. too short, to tell circular from collinear apart and any aligner would naturally fail to reliably predict the circularization.

total	mapped	split-mapped	circular
1119183	1108492 (99%)	504118 (45%)	393212 (35.1%)

Table 6: Results for simulated circularized ALU repeats.

LnCaP split validation

Transcriptome sequencing

Cells from the human prostate carcinoma cell line LNCaP were maintained and total RNA was isolated as described previously [3]. Ribsomal RNAs were removed from total RNA using the Ribo-Zero Kit (Epicentre, Madison, WI) according to the manufacturer's instructions. A strand-specific library for transcriptome sequencing was prepared using the ScripSeq Kit (Epicentre) following the manufacturer's instructions. The library concentration was determined using an Agilent 2100 Bioanalyzer system with a High Sensitivity DNA Kit (Agilent, Sanat Clara, CA) according to the manufacturer's instructions and relying on the concentration of fragments between 150 and 600 nt in size. 12 pmol of library were clustered on one lane of an Illumina paired-end flow cell and 2x100nt were sequenced according to the manufacturer's instructions using the v3 Cluster Generation and SBS Sequencing Kits (Illumina, San Diego, CA) on a HiSeq2000 system.

Split and isoform validation

Reverse transcriptase polymerase chain reaction (RT-PCR) was used to validate the observed split in total RNA of the human carcinoma cell line LNCaP. Complementary DNA was synthesized from $1\mu g$ total RNA using AMV reverse transcriptase (Finnzymes) and random hexamer primers (Invitrogen). PCR was performed using FAST SYBR^{TM}green (Applied Biosystems) on a Applied Biosystems 7900 System with the following primer sets:

```
forward primer 29: 5'-GGTCCCTTTTCTTTGACCAG-3', 300nM
reverse primer 29: 5'-CTGCAGGATCTATAGGCAGCTT-3', 300nM
forward primer 36: 5'-GTCCCTTTTCTTTGACCAGAT-3', 300nM
reverse primer 36: 5'-GGGACAGGATCTATAGGCAGCTT-3', 300nM
```

Primers were designed using primer3 [16] and the sequence of the read exhibiting the split as a template, which explains mismatches in the primer sequences compared to the reference genome. Following PCR and agarose gel electrophoresis, amplified bands (around 100bp) were gel excised (Qiagen MinElute Gel extraction kit) and the purified products were ligated into a pCR4-Topo vector and transformed using TOP10 chemical competent Escherichia coli cells (TOPO TA Cloning Kit, Invitrogen). Clones were checked for inserts with M13F and M13R primers and PCR-products were subjected to Sanger sequencing.

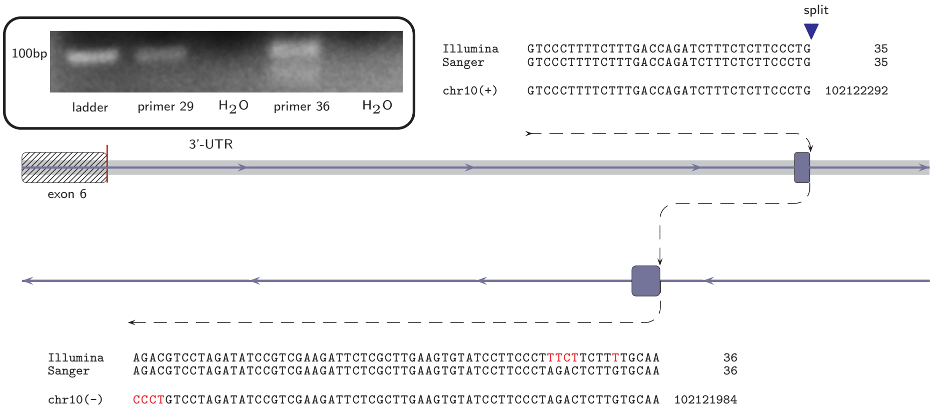

Figure 5: A split observed in the prostate carcinoma cell line LNCaP. The split aligns in parts to the forward and reverse strand of chromsome 10, respectively, and is located in the 3' UTR of stearoyl-CoA desaturase (SCD). This enzyme is known to promote prostate cancer cell proliferation and transactivation of the androgen receptor, the key signaling pathway in this disease [12]. LNCaP total RNA was sequenced strand-specific using Illumina paired-end sequencing subsequent to ribosomal RNA removal. For validation, PCR primers were designed using the sequence reads exhibiting the split as a template. Conventional RT-PCR was performed using these primers and the amplicon of the expected size shown in the inset was cloned. Sanger sequencing of this clone confirmed the observed split.

Recovery of published circular RNAs

The existence of circRNAs has been demonstrated in several high-impact publications over the last couple of years, which are cited in the manuscript. We demonstrate, using the RNA seq data for HEK293 cells from the Memczak et al. (Nature 2013), that segemehl is capable of recovering the circRNAs reported and validated in these authors' work.
In total, segemehl found 1,712 circular junctions located at canonical splice motifs. From the 239 predicted circular junctions in the Memczak paper (cf. [13]), 191 (80%) were also predicted by our algorithm. More importantly, our mapper predicted **all** of the 19 circular RNAs that were experimentally validated by Memczak. In fact, we observed that the majority of the validated RNAs (n=15) had a junction support of two or more reads. Applying this simple filter reduced the number of reported circular junctions to 373.

References

[1] Kin Fai Au, Hui Jiang, Lan Lin, Yi Xing, and Wing Hung Wong. Detection of splice junctions from paired-end RNA-seq data by SpliceMap. *Nucleic Acids Res*, 38:4570–4578, 2010.

[2] Michael F Berger, Joshua Z Levin, Krishna Vijayendran, Andrey Sivachenko, Xian Adiconis, Jared Maguire, Laura A Johnson, James Robinson, Roel G Verhaak, Carrie Sougnez, Robert C Onofrio, Liuda Ziaugra, Kristian Cibulskis, Elisabeth Laine, Jordi Barretina, Wendy Winckler, David E Fisher, Gad Getz, Matthew Meyerson, David B Jaffe, Stacey B Gabriel, Eric S Lander, Reinhard Dummer, Andreas Gnirke, Chad Nusbaum, and Levi A Garraway. Integrative analysis of the melanoma transcriptome. *Genome Res*, 20:413–427, 2010.

[3] K. Boll, K. Reiche, K. Kasack, N. Mörbt, A. K. Kretzschmar, J. M. Tomm, G. Verhaegh, J. Schalken, M. von Bergen, F. Horn, and J. Hackermüller. MiR-130a, miR-203 and miR-205 jointly repress key oncogenic pathways and are downregulated in prostate carcinoma. *Oncogene*, 32:277–285, 2013.

[4] Sarah Djebali, Carrie A. Davis, Angelika Merkel, Alex Dobin, Timo Lassmann, Ali M. Mortazavi, Andrea Tanzer, Julien Lagarde, Wei Lin, Felix Schlesinger, Chenghai Xue, Georgi K. Marinov, Jainab Khatun, Brian A. Williams, Chris Zaleski, Joel Rozowsky, Maik Röder, Felix Kokocinski, Rehab F. Abdelhamid, Tyler Alioto, Igor Antoshechkin, Michael T. Baer, Philippe Batut, Kimberly Bell, Ian Bell, Sudipto Chakrabortty, Xian Chen, Jacqueline Chrast, Joao Curado, Thomas Derrien, Jorg Drenkow, Erica Dumais, Jacqueline Dumais, Radah Duttagupta, Emilie Falconnet, Meagan Fastuca, Kata Fejes-Toth, Pedro Ferreira, Sylvain Foissac, Melissa J. Fullwood, Hui Gao, David Gonzalez, Assaf Gordon, Harsha Gunawardena, Cedric Howald, Sonali Jha, Rory Johnson, Philipp Kapranov, Brandon King, Colin Kingswood, Oscar J. Luo, Eddie Park, Jonathan B. Preall, Kimberly Persaud, Paolo Ribeca, Brian Risk, Daniel Robyr, Michael Sammeth, Lei-Hoon See, Atif Shahab, Lorian Schaffer, Jorgen Skancke, Ana Maria Suzuki, Hazuki Takahashi, Hagen Tilgner, Diane Trout, Nathalie Walters, Huaien Wang, John Wrobel, Yanbao Yu, Xiaoan Ruan, Yoshihide Hayashizaki, Jennifer Harrow, Mark Gerstein, Tim Hubbard, Alexandre Reymond, Stylianos E. Antonarakis, Gregory Hannon, Morgan C. Giddings, Yijun Ruan, Barbara Wold, Piero Carninci, Roderic Guigó, and Thomas R. Gingeras. Landscape of transcription in human cells. *Nature*, 489:101–108, 2012.

[5] Alexander Dobin, Carrie A. Davis, Felix Schlesinger, Jorg Drenkow, Chris Zaleski, Sonali Jha, Philippe Batut, Mark Chaisson, and Thomas R. Gingeras. STAR: ultrafast universal RNA-seq aligner. *Bioinformatics*, 29(1):15–21, 2013.

[6] S. Stricklin et al. *Wormbook*, chapter C. elegans noncoding RNA genes. The C. elegans Research Community, http://www.wormbook.org, 2005.

[7] Gregory R. Grant, Michael H. Farkas, Angel D. Pizarro, Nicholas F. Lahens, Jonathan Schug, Brian P. Brunk, Christian J. Stoeckert, John B. Hogenesch, and Eric A. Pierce. Comparative analysis of RNA-Seq alignment algorithms and the RNA-Seq unified mapper (RUM). *Bioinformatics*, 27(18):2518–2528, 2011.

[8] B. R. Graveley, A. N. Brooks, J. W. Carlson, M. O. Duff, J. M. Landolin, L. Yang, C. G. Artieri, M. J. van Baren, N. Boley, B. W. Booth, et al. The developmental transcriptome of drosophila melanogaster. *Nature*, 471(7339):473–479, 2010.

[9] Lesca M. Holdt, Steve Hoffmann, Kristina Sass, David Langenberger, Markus Scholz, Knut Krohn, Knut Finstermeier, Anika Stahringer, Wolfgang Wilfert, Frank Beutner, Stephan Gielen, Gerhard Schuler, Gabor Gäbel, Hendrik Bergert, Ingo Bechmann, Peter F. Stadler, Joachim Thiery, and Daniel Teupser. Alu elements in anril non-coding rna at chromosome 9p21 modulate atherogenic cell functions through trans-regulation of gene networks. *PLoS Genet*, 9(7):e1003588, 07 2013.

[10] Songbo Huang, Jinbo Zhang, Ruiqiang Li, Wenqian Zhang, Zengquan He, Tak-Wah Lam, Zhiyu Peng, and Siu-Ming Yiu. SOAPsplice: genome-wide ab initio detection of splice junctions from RNA-Seq data. *Frontiers in Genetics*, 2(46), 2011.

[11] Daehwan Kim and Steven Salzberg. TopHat-Fusion: an algorithm for discovery of novel fusion transcripts. *Genome Biology*, 12:R72+, 2011.

[12] Seung-Jin Kim, Hojung Choi, Sung-Soo Park, Chawnshang Chang, and Eungseok Kim. Stearoyl CoA desaturase (SCD) facilitates proliferation of prostate cancer cells through enhancement of androgen receptor transactivation. *Mol Cells*, 31:371–377, 2011.

[13] Rajewski Lab. circBase. 2013.

[14] Tim R. Mercer, Daniel J. Gerhardt, Marcel E. Dinger, Joanna Crawford, Cole Trapnell, Jeffrey A. Jeddeloh, John S. Mattick, and John L. Rinn. Targeted RNA sequencing reveals the deep complexity of the human transcriptome. *Nature biotechnology*, 30:99–104, 2012.

[15] Leorah H. Ross, Jonathan H. Freedman, and Charles S. Rubin. Structure and expression of novel spliced leader rna genes in caenorhabditis elegans. *Journal of Biological Chemistry*, 270(37):22066–22075, 1995.

[16] S. Rozen and H. Skaletsky. Primer3 on the WWW for general users and for biologist programmers. *Methods Mol Biol*, 132:365–386, 2000.

[17] Cole Trapnell, Lior Pachter, and Steven L Salzberg. TopHat: discovering splice junctions with RNA-Seq. *Bioinformatics*, 25:1105–1111, 2009.

[18] Kai Wang, Darshan Singh, Zheng Zeng, Stephen J Coleman, Yan Huang, Gleb L Savich, Xiaping He, Piotr Mieczkowski, Sara A Grimm, Charles M Perou, James N MacLeod, Derek Y Chiang, Jan F Prins, and Jinze Liu. MapSplice: accurate mapping of RNA-seq reads for splice junction discovery. *Nucleic Acids Res*, 38:e178, 2010.

[19] TD Wu and S Nacu. Fast and SNP-tolerant detection of complex variants and splicing in short reads. *Bioinformatics*, 26:873–81, Apr 2010.
